# Calreticulin Induces Dilated Cardiomyopathy

**DOI:** 10.1371/journal.pone.0056387

**Published:** 2013-02-20

**Authors:** Dukgyu Lee, Tatsujiro Oka, Beth Hunter, Alison Robinson, Sylvia Papp, Kimitoshi Nakamura, Wattamon Srisakuldee, Barbara E. Nickel, Peter E. Light, Jason R. B. Dyck, Gary D. Lopaschuk, Elissavet Kardami, Michal Opas, Marek Michalak

**Affiliations:** 1 Department of Biochemistry, University of Alberta, Edmonton, Alberta, Canada; 2 Department of Pediatrics, University of Alberta, Edmonton, Alberta, Canada; 3 Department of Pharmacology, University of Alberta, Edmonton, Alberta, Canada; 4 Department of Laboratory Medicine and Pathobiology, University of Toronto, Toronto, Ontario, Canada; 5 Department of Pediatrics, Kumamoto University School of Medicine, Kumamoto, Japan; 6 Department of Human Anatomy and Cell Sciences, and Physiology and Institute of Cardiovascular Sciences, St. Boniface Research Centre, University of Manitoba, Winnipeg, Canada; University of Western Ontario, Canada

## Abstract

**Background:**

Calreticulin, a Ca^2+^-buffering chaperone of the endoplasmic reticulum, is highly expressed in the embryonic heart and is essential for cardiac development. After birth, the calreticulin gene is sharply down regulated in the heart, and thus, adult hearts have negligible levels of calreticulin. In this study we tested the role of calreticulin in the adult heart.

**Methodology/Principal Findings:**

We generated an inducible transgenic mouse in which calreticulin is targeted to the cardiac tissue using a Cre/loxP system and can be up-regulated in adult hearts. Echocardiography analysis of hearts from transgenic mice expressing calreticulin revealed impaired left ventricular systolic and diastolic function and impaired mitral valve function. There was altered expression of Ca^2+^ signaling molecules and the gap junction proteins, Connexin 43 and 45. Sarcoplasmic reticulum associated Ca^2+^-handling proteins (including the cardiac ryanodine receptor, sarco/endoplasmic reticulum Ca^2+^-ATPase, and cardiac calsequestrin) were down-regulated in the transgenic hearts with increased expression of calreticulin.

**Conclusions/Significance:**

We show that in adult heart, up-regulated expression of calreticulin induces cardiomyopathy *in vivo* leading to heart failure. This is due to an alternation in changes in a subset of Ca^2+^ handling genes, gap junction components and left ventricle remodeling.

## Introduction

Dilated cardiomyopathy, characterized by dilation of the left ventricle (LV) and impaired systolic function, is one of the most common features of cardiac pathology and is exhibited in approximately 60% of all cardiomyopathies. Importantly, dilated cardiomyopathy leads to the development of congestive heart failure in 40 out of 100,000 people and results in high morbidity and mortality. Ca^2+^ is central to cardiac development, physiology and pathology, and Ca^2+^ handling proteins associated with the sarco/endoplasmic reticulum membrane are critical for excitation-contraction coupling in the heart, as well as for housekeeping functions in the cell. The role of membrane-associated Ca^2+^ cycling proteins in dilated cardiomyopathy is not well understood.

In cardiomyocytes, the sarcoplasmic reticulum (SR), a specialized form of the endoplasmic reticulum (ER), plays a key role in the control of excitation-contraction coupling. In the SR, Ca^2+^ is buffered by calsequestrin (CASQ), and is released *via* the ryanodine receptor (RyR) to trigger contraction. Ca^2+^ is taken up by the SR/ER Ca^2+^-ATPase (SERCA) to initiate relaxation [1]. In non-muscle cells, the ER is an intracellular organelle responsible for an assortment of critical cellular functions which include the synthesis, folding, posttranslational modification and transport of proteins; the synthesis of lipids and steroids; and the assembly and trafficking of membranes. The ER is also an important Ca^2+^ storage organelle involved in the regulation of cellular Ca^2+^ homeostasis *via* the action of the inositol 1, 4, 5-triphosphate receptor and SERCA [1]. The discernible distinction between the ER and SR in muscle is still not clear[1]. Recently, ER-associated components and pathways have been implicated in cardiac physiology and pathology [1], [2]. For example, ER stress has been recognized as playing a role in cardiac physiology and pathology [2]. At the early stages of cardiogenesis, ER membranes and ER associated functions dominate in cardiomyocytes and are gradually replaced by highly specialized SR membrane components [1]. Mutation in genes encoding the ER Ca^2+^-buffering chaperones calreticulin and GRP94 affects cardiac development and may play a role in congenital heart diseases [3]–[6].

Calreticulin deficiency is embryonic lethal in mice due to impaired cardiac development, and the protein is highly expressed in embryonic hearts [3], [7]. Similar to other cardiac embryonic genes, following birth cardiac calreticulin is sharply down-regulated and maintained at negligible levels in the adult hearts [8]. However, reactivation of the fetal cardiac gene program is one of the characteristic genetic alterations seen in many cardiac pathologies [9]. Yet, the role of calreticulin in adult heart pathophysiology is not clear.

Here, using transgenic mice, we show that increased expression of calreticulin in the adult heart induces dilated cardiomyopathy and heart failure. Transgenic animals show diminished systolic and diastolic function of the LV, and down-regulation of gap junction proteins and SR-associated Ca^2+^-handling proteins. Calreticulin induced dilated cardiomyopathy in adult hearts is likely due to an alterations in the expression of a subset of Ca^2+^ handling genes, as well as changes in gap junction components and left ventricle remodeling.

## Materials and Methods

### Ethics Statement

All animal experiments were carried out according to the University of Alberta Animal Policy and Welfare Committee and the Canadian Council on Animal Care Guidelines. The approval for use of animals in research was granted by the Animal Care and Use Committee for Health Sciences, a University of Alberta ethics review committee.

### Generation of Transgenic Mice

The transgene vector containing a cytomegalovirus early enhancer/chicken β-actin (CAG) promoter-loxP-CAT gene-loxP-mouse CRT cDNA was used to make CAT-loxP-CRT mice (C57BL/6). The CAT-loxP-CRT mice (“control mice”) were cross-bred with αMHC (myosin heavy chain)-Cre mice (C57BL/6) single time to generate double transgenic mice (designated αMHC/CAT-loxP-CRT) which carried transgenes containing both MerCreMer driven by the αMHC promoter and CAT-loxP-CRT driven by the CAG promoter. To induce calreticulin expression we delivered tamoxifen, which involved feeding mice with tamoxifen mixed into their food. Briefly, 80 mg of tamoxifen was mixed with 200 g of powdered feed in 100 ml of water. Small cakes (8–10 g) were made out of the wet mix, and given to the 8–10 week-old control and αMHC/CAT-loxP-CRT male mice each day for 3 weeks. Tamoxifen induction resulted in excision of the CAT gene and the subsequent production a calreticulin expressing transgene in the heart under the control of the CAG promoter (αMHC/CRT, referred to throughout the paper as “calreticulin transgenic mice”, CRT-TG).

### Echocardiography and Electrocardiography (ECG)

A Vevo-770 (Visualsonics) high-resolution micro-ultrasound system equipped with a 30-MHz probe (RMV^TM^ 707b) was used for transthoracic echocardiography on mice anesthetized with 1.5% isoflurane. Hearts were imaged in the two-dimensional parasternal short-axis view, and a M-mode echocardiogram of the mid-ventricle was recorded. The following measurements were obtained during both systole and diastole: inter-ventricular septal thickness (IVS), left ventricular posterior wall thickness (LVPW), left ventricular internal diameter (LVID), heart rate, ejection fraction (EF). Measurements were averaged from 3 to 6 cardiac cycles according to the American Society of Echocardiography. Percent ejection fraction (%EF) was calculated as follows: 100 * [(end-diastolic volume - end-systolic volume)/end-diastolic volume)]. The Tei index (a measure of myocardial performance) was calculated as the ratio of time intervals (a-b/b), derived by pulsed Doppler echocardiography, where a is the time between the end and the start of transmitral flow, and b is the LV ejection time. For electrocardiography (ECG) measurements, mice were maintained on a heated isothermal plate. ECGs were measured using surface electrode clips, and readings were recorded using Power Lab (ADInstruments) and analyzed by LabChart software (version: 7.3, ADInstruments) [10].

### Real-time PCR and Western Blot Analyses

Total RNA was isolated from hearts using TRIzol reagent (Invitrogen) according to the manufacturer’s instructions. For real-time PCR, a Rotor Gene RG-3000 (Corbett Research) and iQ SYBR Green Supermix (Bio-Rad) were used. The final quantitation of the amount of target (Ct value) in a real-time PCR reaction was converted to the amount of transcript and normalized by glyceraldehyde 3-phosphate dehydrogenase transcript (*Gapdh*). PCR primers used in this study indicated in Text S1.

For Western blot analysis, proteins from various mouse tissues including heart, brain, lung, kidney, and skeletal muscle were lysed, separated by SDS-PAGE, and followed by immunoblotting. Sarcoplasmic reticulum (SR) vesicles were isolated from mouse heart ventricular tissue [11], and SR proteins were separated by SDS-PAGE followed by immunoblotting. Immunoreactive protein bands were detected using peroxidase-conjugated secondary antibodies followed by a standard enhanced chemiluminense reaction [3]. Antibodies used in this study indicated in Text S1.

### Cell Culture and Luciferase Assay

H9C2 (ATCC: CRL-1446), a myoblast cell line, was cultured and maintained in Dulbecco's Modified Eagle Medium (DMEM) containing 10% bovine growth serum (BGS) and 1% penicillin/streptomycin. H9C2 cells were stably transfected with HA-tagged full length calreticulin (*H9C2+CRT*) or the P+C domains of calreticulin containing Ca^2+^ buffering region of calreticulin (*H9C2+CRT-PC*). A 2.3 kb Cx43 promoter region (*Gja1*, NM_010288.3) was isolated from mouse genomic DNA, and subcloned into a pGL3-basic luciferase vector (Promega) to construct pGL3-Cx43. H9C2 cell lines were co-transfected with pSV-β-galactosidase vector (Promega) and pGL3-Cx43 plasmid using Lipofectamine 2000 (Invitrogen). After 48 h, cells were harvested and luciferase activity was measured [12]. Luciferase activity was normalized to β-galactosidase activity. The mean ± SEM of three independent experiments (each in triplicate) is reported.

### Immunohistochemistry

Mice were euthanized by cervical dislocation and excised heart tissues were fixed overnight in 10% formalin buffered with phosphate buffered saline (PBS), paraffin embedded, sectioned at 5 µm and stained with hematoxylin and eosin [13]. For immunofluorescence [14], transverse 7 µm thick cryosections were obtained using a Microm HM550 cryostat [3]. For connexin staining, heart tissue sections were fixed with 1% cold paraformaldehyde (60 sec) followed by permeabilization with 0.1% Triton-X 100-PBS. Sections were incubated overnight with primary antibodies (1∶2000 dilution for anti-Cx43 antibodies) [14]. Antigen-antibody complexes were visualized after incubation with secondary antibodies: biotinylated anti-rabbit IgG (Amersham Biosciences) at 1∶20 dilution in 1% bovine serum albumim/PBS followed by streptavidin-fluorescein (Amersham Biosciences) at 1∶20 dilution. Sections stained with mouse primary antibodies were incubated with Texas Red conjugated-anti-mouse IgG (Jackson Laboratories) at 1∶100 in 1% BSA/PBS. Nuclei were visualized with 2.5 mM Hoechst dye 33342 (Calbiochem). Images were captured on a Zeiss Axiovert 3.0 epifluorescence microscope.

### Statistical Analysis

All data are presented as mean±SEM. Statistical analysis of raw data was performed using Origin8 software (Origin Lab) with the Student paired *t-*test and One-way ANOVA (analysis of variance). Statistical significance was accepted at a *p* value<0.05.

## Results

### Cardiac-specific Induction of Calreticulin Leads to Dilated Cardiomyopathy

**Figure 1:**
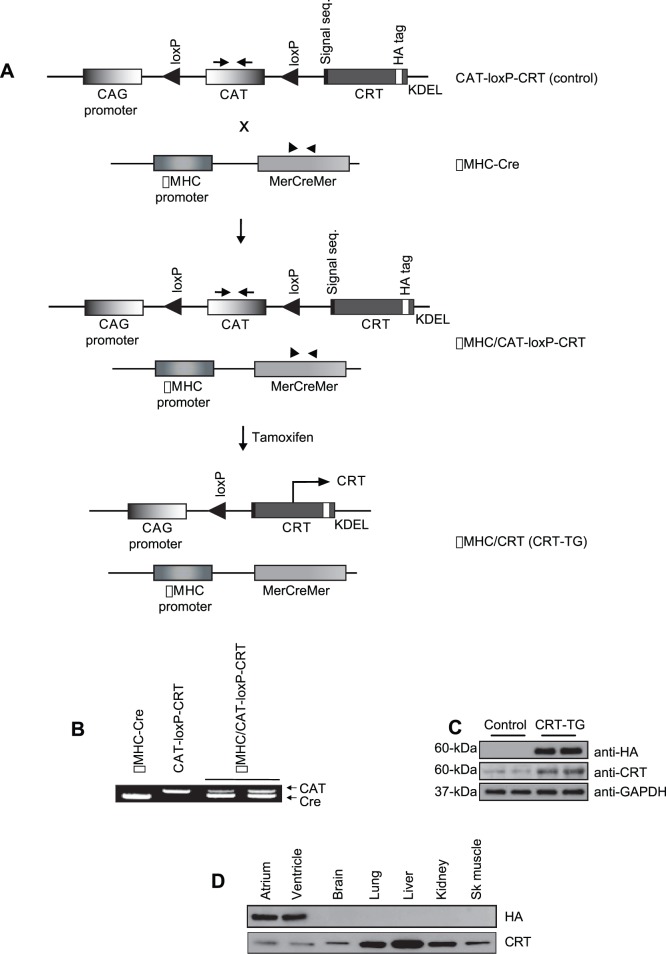
Generation of cardiac-specific and inducible calreticulin transgenic mice. (A) The CAT-loxP-CRT transgene consisted of a CAG promoter, the CAT gene flanked with loxP-sites followed by cDNA encoding HA-tagged calreticulin. Cardiac specific cre mice express cre recombinase (*MerCreMer*) under the control of αMHC promoter (*αMHC-Cre*). Double transgenic mice were generated by breeding CAT-loxP-CRT mice with αMHC-Cre mice to produce αMHC/CAT-loxP-CRT mice. Administration of tamoxifen triggers nuclear translocation of MerCreMer and cardiac-specific Cre mediated recombination and excision of the floxed CAT gene, resulting in the expression of HA-tagged calreticulin under the control of CAG promoter (αMHC/CRT mice). αMHC/CRT mice are referred to throughout the paper as calreticulin transgenic mice (CRT-TG). Arrows and arrow heads indicate the location of primers used for PCR-driven genomic DNA analysis of transgenic mice. The location of loxP sites is indicated. CAG, CMV early enhancer/chicken β actin; HA, hemagglutinin; CAT, chloramphenicol acetyltransferase, Cre, Cre recombinase (type I topoisomerase); αMHC, α-myosin heavy chain; Mer, modified estrogen receptor. CRT, calreticulin; KDEL, ER retention signal sequence. (B) Genotyping of the αMHC-Cre, CAT-loxP-CRT, and αMHC/CAT-loxP-CRT mice was carried out by PCR-driven amplification using primers shown in Figure 1A (arrows for CAT gene and arrow heads for MerCreMer gene). (C) Western blot analysis of expression of recombinant CRT (anti-HA) and endogenous CRT (anti-CRT) after feeding tamoxifen for 3 weeks. Glyceraldehyde 3-phosphate dehydrogenase (anti-GAPDH) was used as a loading control. (D) Tissue specific expression of recombinant calreticulin (*anti-HA*) in CRT-TG mice and expression of endogenous calreticulin (*anti-CRT*) from control mouse. Sk muscle, skeletal muscle.

To generate cardiac-restricted and inducible calreticulin transgenic mice, first we generated a CAT-loxP-CRT mouse harbouring the CAT-loxP-calreticulin transgene that consisted of the CAG promoter followed by a cDNA encoding influenza HA (hemagglutinin)-tagged calreticulin. Next, CAT-loxP-CRT mice were cross-bred with αMHC-Cre mice containing a cardiac-specific MerCreMer transgene (Fig. 1A). The presence of a specific transgene in mice was confirmed by PCR (Fig. 1B). In this system, expression of calreticulin can be induced by administration of tamoxifen to promote nuclear translocation of MerCreMer resulting in excision of the CAT gene and consequently producing a calreticulin expressing transgene under control of the CAG promoter (αMHC/CRT, referred to throughout the paper as “calreticulin transgenic mice”, CRT-TG) (Fig. 1A). Tamoxifen administration via feeding had no detrimental effects on the treated animals, and reproducibly induced the expression of hemagglutinin (HA)-tagged calreticulin in the heart (Fig. 1C). As well, side effects on cardiac function were not observed in tamoxifen-induced transgenic lines carrying αMHC-Cre. Optimal and stable expression of the HA-tagged calreticulin in the heart was achieved 3 weeks post-tamoxifen feeding (Fig. 1D). The recombinant HA-calreticulin was induced only in the heart tissue (atrium and ventricle) of CRT-TG mice, but not in other tissues including brain, lung, liver, kidney, or skeletal muscle (Fig. 1D, upper panel). Immunoblotting with goat anti-calreticulin antibody of various tissues from CAT-loxP-CRT (control mice) to detect endogenous calreticulin showed that the protein was expressed in all tissues with a relatively high level in the liver and lung (Fig. 1D, lower panel).

**Figure 2:**
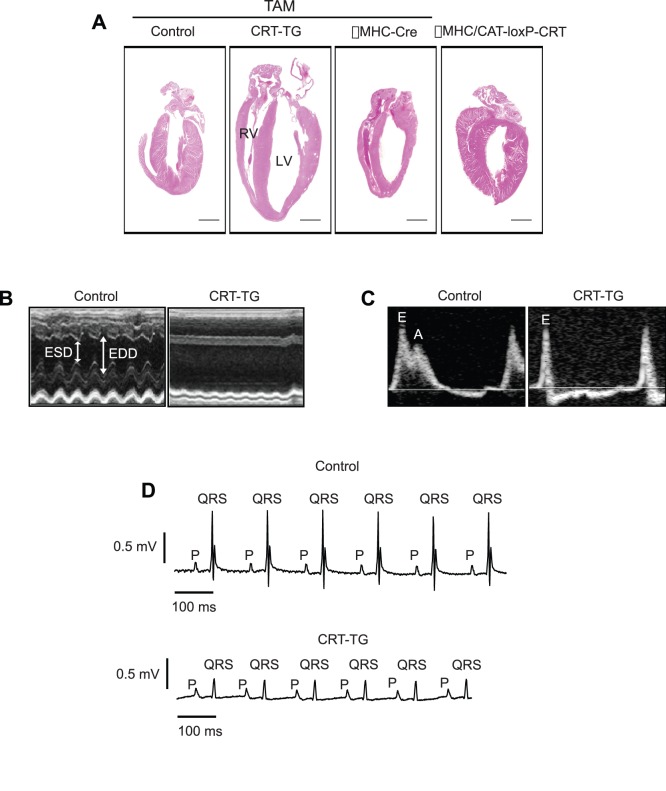
Gross morphology, echocardiography and electrocardiographic presentations of hearts with increased expression of calreticulin. (A) Hematoxylin and eosin staining of hearts from control (CAT-loxP-CRT), αMHC-Cre and CRT-TG mice fed tamoxifen for 3 weeks. Histological analysis of αMHC/CAT-loxP-CRT double transgenic not fed tamoxifen is also included. Scale bar, 1 mm. TAM, tamoxifen; LV, left ventricle; RV, right ventricle. (B) Representative M-mode echocardiography images of control (*CAT-loxP-CRT*) and CRT-TG hearts from mice fed tamoxifen for 3 weeks. ESD, end systolic diameter; EDD, end diastolic diameter. (C) Representative images of transmitral flow velocity pattern in the pulmonary venous flow in control and CRT-TG hearts. E, E-wave; A, A-wave. (D) Representative electrocardiography recording images of hearts from control and CRT-TG mice fed tamoxifen for 3 weeks (n = 10).

There were no differences in survival rate between tamoxifen induced transgenic mice and αMHC/CRT-loxP-CRT mice. These animals also did not show any noticeable behavioral differences. As early as 1 week after the induction of calreticulin expression less 30% of CRT-TG mice revealed reduced %EF and dilation of LV chamber. Histological analysis showed that adult hearts with increased expression of calreticulin exhibited an enlarged LV chamber with thinned posterior wall (Fig. 2A). In contrast, tamoxifen fed control (CAT-loxP-CRT) and αMHC-Cre, or not fed αMHC/CRT-loxP-CRT mice did not show any abnormal heart morphology (Fig. 2A). The ratio of LV mass to body weight of CRT-TG mice was increased compared to control mice with tamoxifen induction (Table 1).

**Table 1:** Echocardiography of control and CRT-TG mice.

Measurement	Unit	Control(n = 15)	CRT-TG(n = 18)	Significance
Body Weight	g	28.6±0.76	22.9±0.84	**
Heart Rate	bpm	495.6±53.7	549.4±61.0	ns
**LV dimensions and functions**				
IVSd	mm	0.73±0.01	0.66±0.03	*
LVIDd	mm	4.07±0.06	4.97±0.10	**
LVPWd	mm	0.74±0.01	0.58±0.03	**
%EF		64.5±2.0	30.8±4.35	**
LV mass/body weight	mg/g	3.01±0.16	4.14±0.31	*
Mitral inflow				
E velocity	mm/sec	708.73±41.2	636.92±48.9	*
ME/A ratio	mitral	1.50±0.03	na	–
Tei index		0.57±0.0	0.91±0.09	**
**Pulmonary vein flow**				
D wave	mm/sec	655.16±48.1	310.04 + 51.25	**
Ar durationtime	msec	8.7±1.9	27.52±2.3	**

Numbers are mean±SEM. LV, left ventricle; IVSd, intraventricular septum diastolic; LVIDd, left ventricle inner diameter diastolic; LVPWd, left ventricle posterior wall diastolic; %EF, percentage of ejection fraction; Tei index, an index of myocardial performance in systolic and diastolic function; Ar, artrial reverse. Statistical significance, *p<0.05 and **p<0.01. ns, not significant. na, not available.

Next we carried out M-mode non-invasive transthoracic echocardiography analysis of tamoxifen induced CAT-loxP-CRT (control) and CRT-TG adult hearts (Movie S1). Figure 2B shows that the CRT-TG heart with increased expression of calreticulin showed severe LV dilation. Hearts of CRT-TG animals exhibited heart rate similar to control mice (Table 1). The systolic function of the LV, represented by ejection fraction (%EF), was significantly decreased (Table 1), and the Tei index of the LV, an indicator of systolic and diastolic function, was increased in CRT-TG hearts (Table 1) after 3 weeks of tamoxifen administration. Moreover, a significant increase in LVID was observed in CRT-TG hearts with calreticulin induction, whereas CAT-loxP-CRT control hearts displayed a normal range for LVID (Table 1). The higher value of the Tei index in CRT-TG hearts versus control hearts was attributable to a prolongation of the isovolumic intervals and a shortening of EF (Table 1). Interestingly, CRT-TG hearts also showed a highly restricted pattern of transmitral flow velocity as evidenced by the absence of an A-wave (Fig. 1C). The increment of the atrial reverse (Ar) wave duration time in the pulmonary venous flow indicated an elevation in LV end-diastolic pressure (Table 1). Severe dysfunction of diastolic function in CRT-TG hearts was demonstrated by reduced E velocity, undetermined A velocity of mitral inflow, and a prolonged duration time from pulmonary vein to the LV. In summary, up-regulation of the calreticulin gene in the adult heart resulted in rapid development of dilated cardiomyopathy and heart failure.

Dilated cardiomyopathy can cause conduction delays and arrhythmias, both of which can be detected using ECG analysis. Therefore, we carried out ECG analysis on transgenic mice, which indicated a different heart rate in CRT-TG mice as seen by echocardiography (Table 1). The P wave amplitude as a proportion of the QRS amplitude was increased in the CRT-TG hearts, indicative of enlargement of the cardiac chambers (Fig. 2D). The QRS amplitude was also significantly reduced in the CRT-TG hearts (0.306±0.003 mV) compared to control hearts (0.940±0.012 mV) (Fig. 2D). There was also weak T amplitude of electrical impulse [0.012±0.015 mV (CRT-TG) versus 0.42±0.005 mV (control)] (Fig. 2D). Further analysis of ECGs from CRT-TG mice showed a longer PR interval indicative of prolonged AV conduction (Fig. 2D). Taken together, the ECG analysis further supported our conclusion that up-regulation of calreticulin in adult heart leads to dilated cardiomyopathy and a significant impairment of systolic and diastolic function.

### Alteration of SR Ca^2+^-handling Proteins in αMHC/CRT Hearts

**Figure 3:**
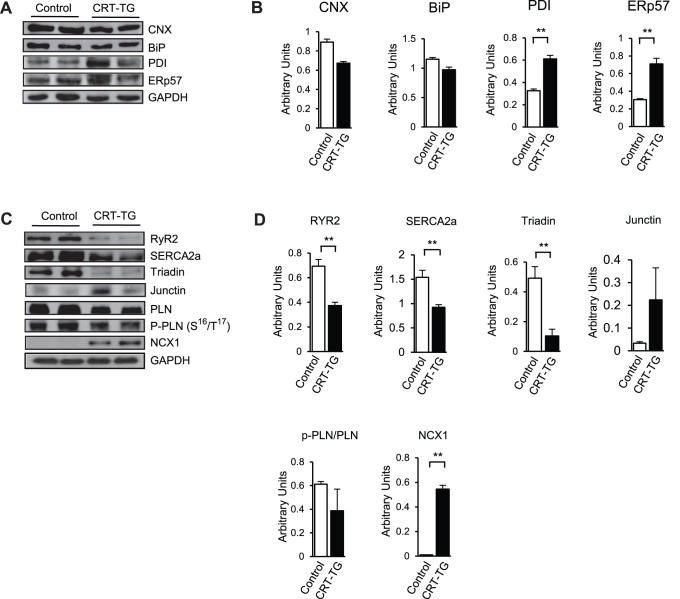
Attenuation of sarcoplasmic reticulum associated proteins in hearts of CRT-TG mice. (A) Cardiac tissues from control and CRT-TG mice fed tamoxifen for 3 weeks were harvested, lysed followed by SDS-PAGE, transferred to nitrocellulose membrane, and probed with specific antibodies. Anti-GAPDH antibodies were used as a loading control. CNX, calnexin; PDI, protein disulfide isomerase. (B) Quantitative analysis of Western blots of sarcoplasmic reticulum associated proteins. **p<0.01 (n = 4). (C) Western blot analysis of SR-associated proteins from control and CRT-TG hearts. SR membrane vesicles were isolated from hearts of control and CRT-TG mice fed tamoxifen for 3 weeks followed by SDS-PAGE and Western blot analysis with specific antibodies. RYR2, cardiac ryanodine receptor; SERCA2a, cardiac sarcoplasmic/endoplasmic reticulum Ca^2+^ ATPase; PLN, phospholamban, p-PLN(S^16^/T^17^), phospho-phospholamban at Ser^16^/Thr^17^ amino acid residues, NCX1, sodium/calcium exchanger-1. GAPDH was used as a loading control. (D) Quantitative analysis of SR-associated proteins from control and CRT-TG hearts. **p<0.01 (n = 4). For junctin quantitative analysis in *D*: *p*>0.25.

Calreticulin is a Ca^2+^ buffering chaperone of the ER and an important component of the quality control of the protein secretory pathway. Therefore, we next analyzed whether increased expression of calreticulin in adult heart had any effect on the molecular make-up of the proteins in the ER and SR membranes of the heart. Western blot analysis of hearts from control and CRT-TG mice fed tamoxifen showed that expression of calnexin, a type I integral ER transmembrane chaperone similar to calreticulin, was slightly decreased in transgenic hearts (Fig. 3A,B). Expression of BiP/GRP78, a heat shock protein 70-kDa family member, was similar in control and CRT-TG hearts (Fig. 3A,B). Interestingly, the expression of two ER associated thiol oxidoreductases, PDI and ERp57, was increased in CRT-TG hearts (Fig. 3A,B).

Next, we focused on the SR-associated proteins involved in Ca^2+^ cycling, the central coordinator of cardiac contraction and relaxation. SR membrane fractions were isolated from the hearts of control and CRT-TG mice fed tamoxifen followed by Western blot analysis. The expression of SR associated Ca^2+^-handling proteins was decreased in transgenic hearts, including the cardiac ryanodine receptor (RYR2), SR/ER Ca^2+^ ATPase (SERCA2a), and triadin (Fig. 3C,D). The non-phosphorylated as well as the phosphorylated (Ser^16^/Thr^17^) form of phospholamban showed a decreased expression in the CRT-TG hearts (Fig. 3C,D). Interestingly, the sodium/calcium exchanger 1 (NCX1) and junctin proteins were increased in hearts from CRT-TG mice (Fig. 3C,D). Figure 3C shows that increased expression of junctin appeared to be inconsistent and to very for different transgenic hearts tested. Most importantly, statistical analysis of several Western blots indicated that there was no significant difference in the level of junctin between CRT-TG and control hearts (Fig. 3D). Taken together these observations suggest that increased expression of calreticulin in the adult heart induces molecular “re-modeling” of the SR-associated Ca^2+^ handling membrane network. This may, in part, provide a molecular explanation for the dilated cardiomyopathy and heart failure seen in the CRT-TG mice.

**Figure 4:**
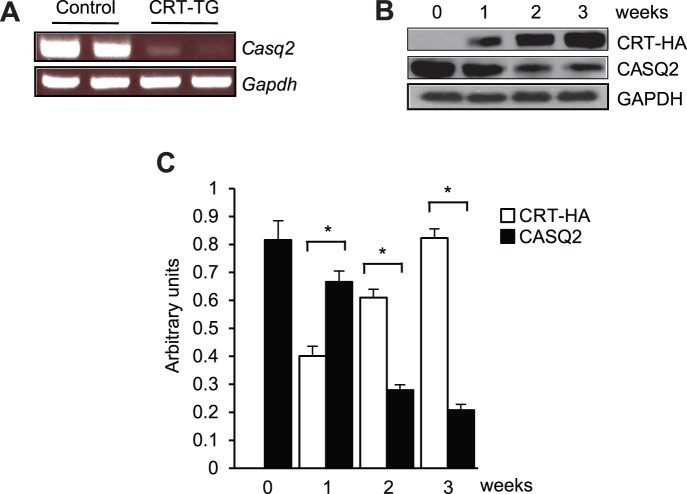
Down-regulation of calsequestrin transcript and protein in hearts from CRT-TG mice. (A) Semi-quantitative RT-PCR analysis of calsequestrin (CASQ2) in hearts isolated from control and CRT-TG mice fed tamoxifen. Gapdh, glyceraldehyde 3-phosphate dehydrogenase. (B) Western blot analysis with anti-HA (*CRT-HA*, for recombinant calreticulin) and anti-CASQ2 antibodies. Control and CRT-TG mice were fed tamoxifen for 1, 2 and 3 weeks, following which hearts were harvested and processed for Western blot analysis. GAPDH was used as a loading control. HA, hemagglutinin; CASQ2, cardiac calsequestrin. (C) Quantitative analysis of expression of CASQ2 in mice with induced expression of calreticulin in adult heart. (mean±SEM; n = 3). CASQ2, cardiac calsequestrin; CRT-HA, calreticulin hemagglutinin. Quantitation of CRT-HA and CASQ2 expression depicted in (B) *p<0.05 (n = 3).

Calsequestrin and calreticulin are two major muscle and non-muscle Ca^2+^ buffering proteins, respectively [1]. In the adult heart, calsequestrin is considered the major Ca^2+^ buffering protein responsible for the storage of over 90% of the SR luminal Ca^2+^ [1]. We observed a remarkable down-regulation of the CASQ2 transcript (Fig. 4A) and protein (Fig. 4B) in hearts with increased expression of calreticulin. Figures 4B,C show that increased expression of recombinant calreticulin was seen in CRT-TG hearts as early as the first week of tamoxifen feeding and, with an even greater decrease after 3 weeks of feeding. A concomitant sharp decrease in the expression of CASQ2 was observed between week 1 and 3 of tamoxifen feeding of CRT-TG mice (Fig. 4B,C). When calreticulin expression was the highest, the expression of CASQ2 was decreased by over 75% compared with that of the control hearts (Fig. 4C). In summary, expression of the ER associated Ca^2+^ buffer calreticulin led to down-regulation of expression of SR associated Ca^2+^buffer, calsequestrin.

**Figure 5:**
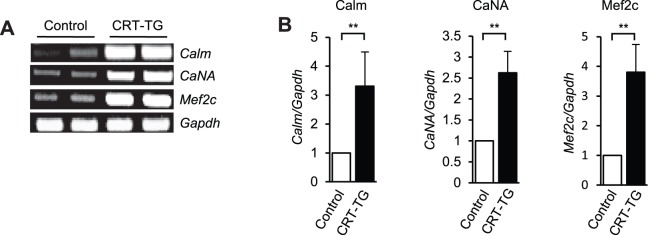
Ca^2+^ signaling molecules in hearts from CRT-TG mice. (A) Semi-quantitative RT-PCR analysis of mRNA encoding Ca^2+^ signaling genes in control and CRT-TG animals. Calm, calmodulin; CaNA, calcineurin A; MEF2c, myocyte enhancing factor 2c; GADPH, glyceraldehyde 3-phosphate dehydrogenase. (B) Quantitative analysis of RT-PCR of mRNA encoding Ca^2+^ signaling genes. **p<0.01 (n = 4).

Calreticulin has been implicated in the activation of many Ca^2+^ signaling molecules including MEF2c, myocyte enhancer factor 2c, transcription factor and calcineurin, a Ca^2+^/calmodulin-dependent protein phosphatase [15]. Therefore, we investigated whether expression of these Ca^2+^ signaling molecules may be modified in hearts of the CRT-TG mice and, therefore, contribute to the pathology of CRT-TG mice. The expression of calmodulin, calcineurin and MEF2c were increased in calreticulin double transgenic hearts (Fig. 5). These findings indicate that in addition to changes in membrane associated Ca^2+^ buffering proteins, increased expression of calreticulin affects cytoplasmic Ca^2+^-dependent transcriptional signaling pathways.

### Reduced Levels of Connexins in Calreticulin Transgenic Hearts

**Figure 6:**
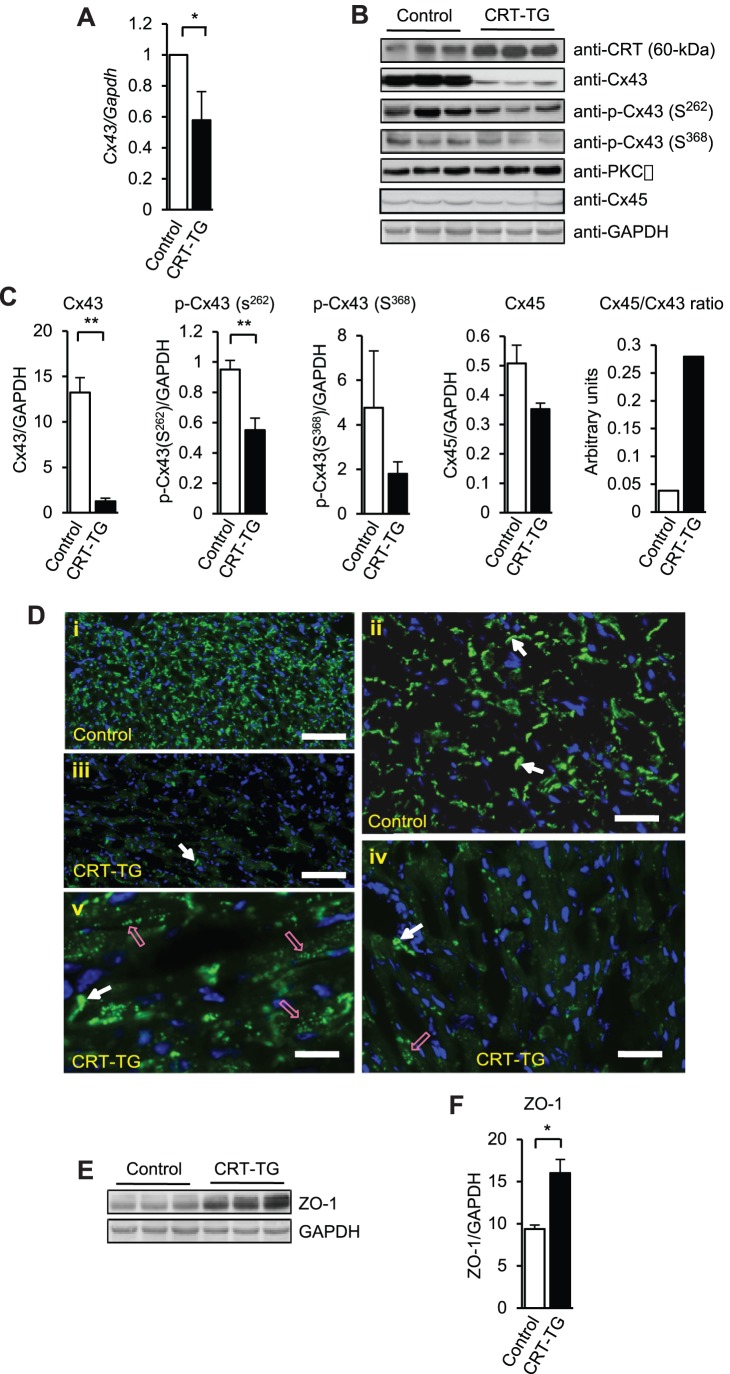
Reduced expression of connexins in hearts from CRT-TG mice. (A) Real-time Q-PCR analysis of Cx43 transcript level in control and CRT-TG hearts. *p<0.05 (n = 5). (B) Western blot analysis of hearts from control and CRT-TG mice probed with anit-CRT, anti-Cx43, anti-phospho-Cx43(S^262^), anti-phospho-Cx43(S^368^), anti-PKCε, and anti-Cx45 antibodies. GAPDH was used as a loading control. HA, hemagglutinin; PKCε, protein kinase C-epsilon. (C) Quantitative analysis of the level of Cx43, p-Cx43(S^262^), phospho-Cx43(S^368^), Cx45, and Cx45/Cx43 ratio. **p<0.01 (n = 3). (D) Distribution pattern of Cx43 gap junctions in ventricular myocardium in control (*i, ii*) and CRT-TG mice (*iii, iv, v*). *i, ii*, longitudinal sections from control hearts, at low (*i*) and high magnifications, (*ii*). The arrows in *ii* indicate a regular pattern of Cx43 intercalated disk staining. Scale bars, 200 µm (*i*) and 100 µm (*ii*). *iii, iv, v*, longitudinal sections from CRT-TG hearts, at low (*iii*) and high (*iv, v*) magnifications. In *iii*, Cx43 patchy staining with areas of near complete lack of Cx43. In *iv*, enlarged view of an area with a weak anti-Cx43 staining. In *v*, areas of disorganized Cx43 punctate staining (*open pink arrows*). The arrows indicate a regular pattern of Cx43 intercalated disk staining. Scale bars: 200 µm for *iii,* 100 µm for *iv* and 50 µm for *v*. (E) Western blot analysis of hearts from control and CRT-TG mice probed with anti-ZO-1 antibodies. GAPDH was used as a loading control. ZO-1; zona occludens-1. (F) Quantitative analysis of ZO-1 expression. *p<0.05 (n = 3).

Dilated cardiomyopathy is frequently associated with pathological changes in cardiac conduction and the ECGs of CRT-TG hearts revealed slightly longer PR intervals (Fig. 2D) indicative of an AV disturbance. Therefore, we next examined expression and intracellular localization of the gap junction connexin (Cx43 and Cx45) proteins. Quantitative PCR analysis showed that expression of Cx43 mRNA was significantly reduced in hearts from CRT-TG mice fed tamoxifen (Fig. 6A). There was also a significant, several fold decrease in Cx43 protein level in the CRT-TG hearts (CRT-TG, 1.26±0.36 versus control, 13.23±1.64) (Fig. 6B,C). Next, we examined the intracellular distribution of Cx43 in control and CRT-TG hearts. As expected, the distribution of Cx43 followed a regular pattern of intercalated disk in control hearts (Fig. 6D, *i, ii*). In contrast, in CRT-TG hearts there were significant large areas that exhibited a large reduction or complete lack of Cx43 staining (Fig. 6D, *iii, iv, v*). This was in agreement with the low expression of Cx43 in the CRT-TG mice (Fig. 6A–C). In addition, Cx43 was also localized away from intercalated disks, in lateral cardiomyocyte surfaces.

Cx43 phosphorylation regulates many of the gap junction properties with respect to assembly, turnover, electrical coupling, and trafficking [16]. For example, Cx43 phosphorylation at S^262^ and S^368^ mediates cardioprotection [14]. Therefore, we examined the level of phospho-Cx43(S^262^) and phospho-Cx43(S^368^) using specific antibodies. Levels of the phosphorylated S^262^ Cx43 [P-Cx43(S^262^)] and S^368^ Cx43 [P-Cx43(S^368^)] were also reduced in the CRT-TG hearts (Fig. 6B,C). Next we tested the expression of Cx45 in transgenic hearts. Figure 6B shows that expression of Cx45 was also reduced in CRT-TG hearts. Although Cx45 is not abundant cardiac connexin, it can form hetero-typic gap junctions with Cx43, where Cx45 plays a dominant effect (decreasing conductance)[16]. Therefore, the ratio of Cx45/Cx43 is an important indicator of gap junction properties. Figure 6C shows that Cx45 was the dominant connexin in CRT-TG hearts as the ratio of Cx45/Cx43 was increased in the CRT-TG hearts, consistent with increased incidence of abnormal conduction. As expected, control and αMHC/CRT mice not receiving tamoxifen had a similar pattern of protein expression to that seen in the tamoxifen fed control mice (Fig. S1).

Zona occludens-1 (ZO-1) forms complexes with Cx43 and plays an important role in the stability and remodeling of gap junctions [17], [18]. Therefore, we examined the expression of ZO-1 protein in control and transgenic hearts. Western blot analysis of hearts from control and CRT-TG mice indicated a significant increase in ZO-1 protein (CRT-TG, 16.00±1.61 vs. control, 9.39±0.45) (Fig. 6 E,F).

### Calreticulin Represses Connexin Promoter Activity

**Figure 7:**
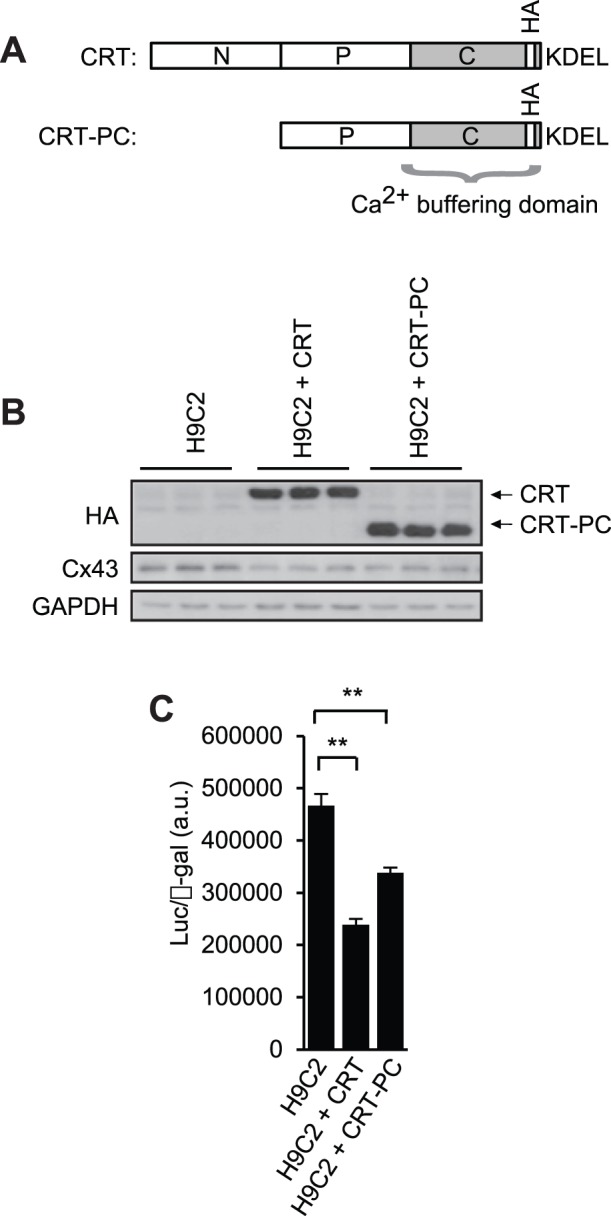
Suppression of Cx43 promoter by calreticulin. (A) A schematic representation of calreticulin and calreticulin domains. CRT, calreticulin full length tagged with HA epitope; CRT-PC, P+C domain of calreticulin tagged with HA epitope. HA, hemagglutinin; KDEL, ER retrieval amino acid sequence. (B) Western blot analysis with anti-HA antibodies (reporting recombinant calreticulin and calreticulin PC domain) and anti-Cx43 antibodies of cell lysates from H9C2 cells (*H9C2*), H9C2 cells expression full length calreticulin (*H9C2+CRT*), and H9C2 cells expression Ca^2+^ buffering PC domain of calreticulin (*H9C2+CRT-PC*). HA, hemagglutinin; CRT, calreticulin; CRT-PC, calreticulin P+C domain. GAPDH was used as a loading control. (C) Control H9C2 cells (*H9C2*), H9C2 cell lines expression full length calreticulin (*H9C2+CRT*) or H9C2 cells expression Ca^2+^ buffering PC domain of calreticulin (*H9C2+CRT-PC*) were transfected with luciferase reporter vector under control of the Cx43 promoter and β-galactosidase expression vector. Cell lysates were harvested and assayed for luciferase activity. **p<0.01 (n = 9).

Calreticulin is composed of distinct structural and functional domains (Fig. 7A)[19]. The C-terminal region of calreticulin plays a role in Ca^2+^ buffering and influences cellular and organellar Ca^2+^ homeostasis [19], [20]. We asked, therefore, if calreticulin’s function as a Ca^2+^ buffering protein affects the expression of connexins. To test this we used H9C2 cells, a myoblast cell line, that was stably transfected with expression vectors encoding either full length calreticulin or the P+C domain (containing Ca^2+^ buffering domain [19]) of the protein (Fig. 7B). The cells were transfected with luciferase reporter plasmid pGL3-Cx43. In the pGL3-Cx43 plasmid, the luciferase reporter gene is controlled by the 2.3 kb Cx43 promoter region. β-galactosidase was used as an internal control. Luciferase activity was significantly reduced in cells with increased expression of calreticulin (Fig. 7C, *H* *9C2+CRT*). Thus, it appears that increased expression of calreticulin in H9C2 cells can suppress transcription from the Cx43 promoter. This is in agreement with a reduced expression of Cx43 in hearts from the CRT-TG mice (Figure 6). Next we transfected H9C2 cells expressing the domain of calreticulin containing Ca^2+^ buffering region (Fig.7A,B) with the pGL3-Cx43 vector (the luciferase gene controlled by the Cx43 promoter). Figure 7C shows that expression of the Ca^2+^ buffering region of calreticulin (a domain that does not support protein folding activity of calreticulin[19]) also repressed transcription from the Cx43 promoter. We concluded that increased expression of full length calreticulin or its Ca^2+^ domain represses transcription from the Cx43 promoter in the myoblast cell line, H9C2.

## Discussion

We showed here that calreticulin, an ER associated Ca^2+^ buffering chaperone, is capable of inducing dilated cardiomyopathy *in vivo*. Previous studies indicated increased expression of calreticulin in cardiac hypertrophy [11], [21]–[23] and in the human failing heart [24], but (to our knowledge) a role for the protein in inducing dilated cardiomyopathy has not been reported. The calreticulin transgenic hearts exhibit a significant LV dilation concomitant with decreased systolic and diastolic function. Of interest is there was reduced level of SR associated Ca^2+^ handling proteins (“remodeling” of SR membrane) and Ca^2+^ signaling proteins in the transgenic hearts. Our findings indicate that changes in ER Ca^2+^ buffer calreticulin (ER Ca^2+^ homeostasis) in adult heart may lead to cardiac pathology. This is likely due to an alternation in a subset of Ca^2+^ handling genes, connexin proteins and LV remodeling.

The data presented here show calreticulin-dependent alterations of the Ca^2+^ handling components of the SR. For decades, the SR membrane in the heart has been generally accepted as a critical component of excitation-contraction coupling, and the SR has always been considered just a different name for the ER in muscle tissues. However, early molecular studies on the ER proteins, calreticulin and GRP94, has revealed that the ER membrane plays a critical role during cardiac development and in the control of intracellular Ca^2+^ homeostasis in the developing and adult heart [1], [3], [4], [6], [25]. More recently, the role of ER associated functions has been recognized as a potential contributor to cardiac physiology and pathology [2], [26]–[32]. Expression of the SR-associated RyR, SERCA, triadin and calsequestrin are significantly reduced in parallel with up-regulation of ER-associated calreticulin, and these likely impacts on the regulation of excitation-contraction coupling in the heart, thereby contributing to calreticulin-induced dilated cardiomyopathy. It is interesting that reduction of calsequestrin mRNA was greater than that of calsequestrin protein which may be due to a relatively slow turnover of calsequestrin protein. The most profound changes were observed in the expression of calsequestrin, a Ca^2+^ buffering protein and muscle analog of calreticulin [33]. Increased calreticulin protein, and thus increased Ca^2+^ capacity/buffering in the ER in the adult heart leads to a rapid progression to dilated cardiomyopathy and heart failure. In contrast, an increase in calsequestrin expression does not induce dilated cardiomyopathy and does not impact on expression of calreticulin [34]. These observations indicate that calreticulin and increased ER, but not SR Ca^2+^ capacity in the heart have detrimental consequences on the development of cardiac pathology.

One of the major functions of calsequestrin is to define the size of the releasable SR Ca^2+^ pool [35] and therefore, the protein affects Ca^2+^ signals and contractile force. Calsequestrin may also act as a Ca^2+^ sensor which influences triadin/junctin-induced RyR activation at different SR luminal Ca^2+^ concentrations [36]–[38]. At present, it is not clear how this may affect transcriptional regulation of the calsequestrin gene. Taken together, this suggests that expression of calsequestrin in the adult heart might be tightly regulated by its non-muscle analog, ER-associated Ca^2+^ binding protein, calreticulin.

Connexin43 is the major gap junction protein of working cardiomyocytes. Altered gap junction organization and connexin expression are key contributors to rhythm disturbances and contractile dysfunction in heart failure [39], dilated cardiomyopathy [40], [41] or end stage heart failure [42]. It has been estimated that a 70–90% reduction in cardiac Cx43 expression is required to elicit significant arrhythmogenesis [43]. The expression of Cx43 (including S^262^ and S^368^ Cx43) and Cx45 is significantly reduced in calreticulin transgenic hearts with many areas of the heart showing a nearly complete absence of Cx43 and some areas of weak staining. Importantly, a normal Cx43 expression has been shown to be important for baseline, as well as inducible, cardiomyocyte resistance to injury [44]. Decreased Cx43 levels, as observed here, would be expected to result in enhanced vulnerability of the heart to injury or stress.

Connexin45, a far less abundant cardiac connexin [45], can form hetero-typic gap junctions with Cx43, acting in a dominant fashion to disrupt conductance. It is therefore of note that the relative ratio of Cx45/Cx43 was increased in the CRT-TG mice, suggesting increased incidence of heterotypic Cx43/45 channels, and therefore increased gap junction dysfunction. Cx43 is phosphorylated at multiple sites in the normal heart [46]. Of these Cx43 sites, phosphorylation of PKCε targets the S^262^ and S^368^ is implicated in PKCε-mediated cardioprotection [14]. Relative levels of phospho-Cx43(S^262^) in CRT-TG mice were reduced by 42% compared to controls, suggestive of increased cardiac vulnerability to injury. The scaffold protein ZO-1 interacts with Cx43 and regulates gap junction formation and size [18]. Heart failure is associated with increased ZO-1 expression [18], similar to that observed in CRT-TG mice. An increased interaction of Cx43 with ZO-1 in failing hearts has been proposed to contribute to aberrant gap junction remodeling [18]. Taken together, the substantial reduction in Cx43, including phospho-Cx43(S^262^), its heterogeneous distribution, the increased likelihood of heterotypic Cx43/Cx45 channel formation, and increased levels of ZO-1 contribute to aberrant gap junctional remodeling, and increased fragility in the CRT-TG hearts, leading to calreticulin-dependent ECG abnormalities and cardiomyopathy. Molecular pathways responsible for calreticulin-dependent effects on connexins remain to be elucidated. Here we show that, in the H9C2 myoblast cell line, calreticulin (or the calreticulin Ca^2+^ buffering domain) inhibits transactivation of the Cx43 promoter in *in vitro* system. Calreticulin’s function as a modulator of Ca^2+^ homeostasis likely plays a key role in the transcriptional control of the Cx43 gene and, thus in the pathology of the transgenic mice. Calreticulin, *via* its ability to modulate Ca^2+^ homeostasis, affects a number of transcriptional processes including the glucocorticoid receptor [47], androgen receptor [48], NF-AT [8], and MEF2c [15]. Similar to CRT-TG, mice with up-regulation of the MEF2c transcription factor in adult heart also show dilated cardiomyopathy [49]. Interestingly, cardiac specific overexpression of calcineurin in mouse heart leads to an eccentric hypertrophy, dilation and cardiac dysfunction similar to the CRT-TG hearts [50].

In summary, we show that calreticulin induces dilated cardiomyopathy in the adult heart in transgenic mice, and induces “remodeling” of the SR Ca^2+^-cycling proteins and gap junctions. Our findings indicate that ER-dependent (calreticulin-dependent) regulation of Ca^2+^ homeostasis is critical for proper physiological function in the heart. ER membrane components, including calreticulin, may represent novel targets for development of pharmacological compounds to intervene with induction of dilated cardiomyopathy and remodeling of Ca^2+^-handling mechanisms in the heart.

## Supporting Information

Figure S1 **Western blot analysis using HA, CRT, CASQ2, and CX43 antibodies from both control and αMHC/CAT-loxP-CRT heart without tamoxifen and control and αMHC/CRT heart with tamoxifen administration.** GAPDH was used as a loading control. HA, hemagglutinin (detect HA-tagged exogenous calreticulin); CRT, calreticulin; CASQ2, cardiac calsequestrin; Cx43, connexin 43.
(TIF)

Movie S1 **Heart beating pattern of control (sham) and double transgenic (** ***CRT-Tg*** **) hearts after 3 weeks induction of calreticulin.**
(WMV)

Text S1 **Supporting Information.**
(DOCX)
