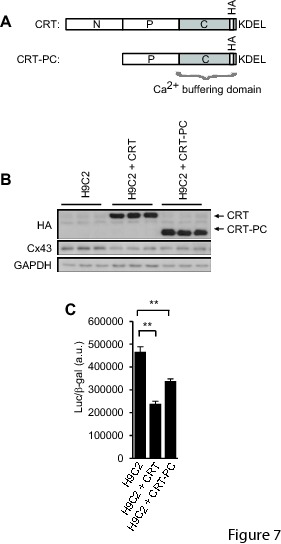# Correction: Calreticulin Induces Dilated Cardiomyopathy

**DOI:** 10.1371/annotation/04c791d5-a71c-493d-b94a-a57b158c5538

**Published:** 2013-11-07

**Authors:** Dukgyu Lee, Tatsujiro Oka, Beth Hunter, Alison Robinson, Sylvia Papp, Kimitoshi Nakamura, Wattamon Srisakuldee, Barbara E. Nickel, Peter E. Light, Jason R. B. Dyck, Gary D. Lopaschuk, Elissavet Kardami, Michal Opas, Marek Michalak

In figure 1A □MHC should be replaced with αMHC in all 6 places. Please see the corrected figure 1 here: 

**Figure:**
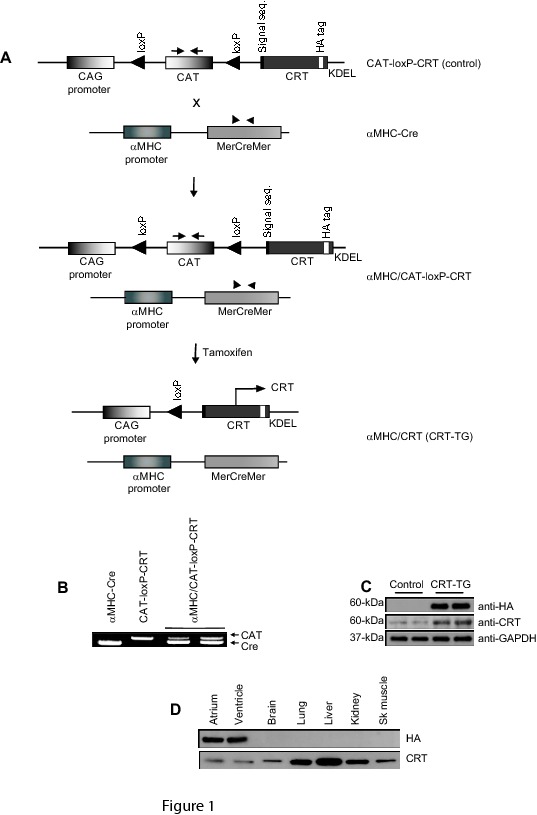


In figure 1B □MHC should be replaced with αMHC in 2 places. Please see the corrected figure 1 here: 

**Figure:**
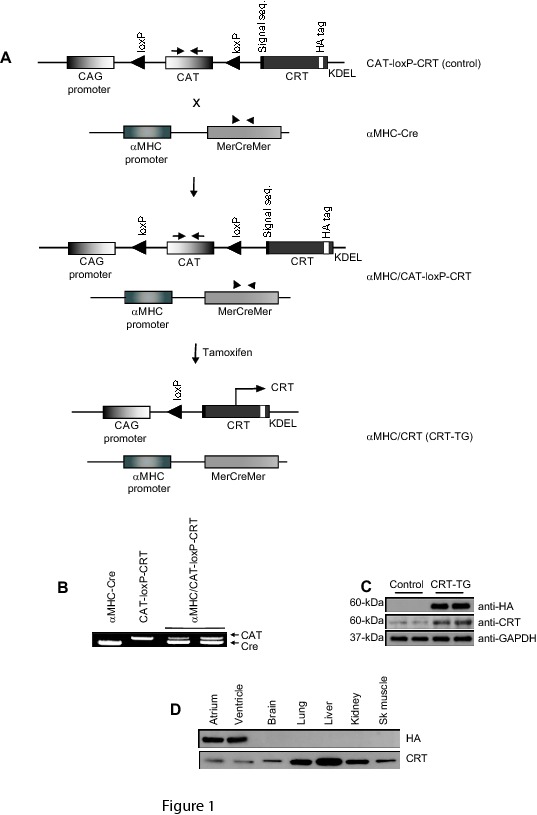


In figure 2A □MHC should be replaced with αMHC in 2 places. Please see the corrected figure 2 here: 

**Figure:**
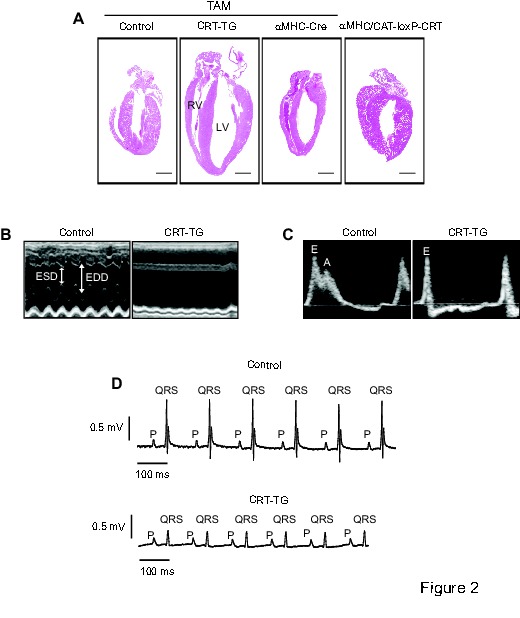


In figure 6B PKC□ should be replaced with PKCε in 1 place. Please see the corrected figure 6 here: 

**Figure:**
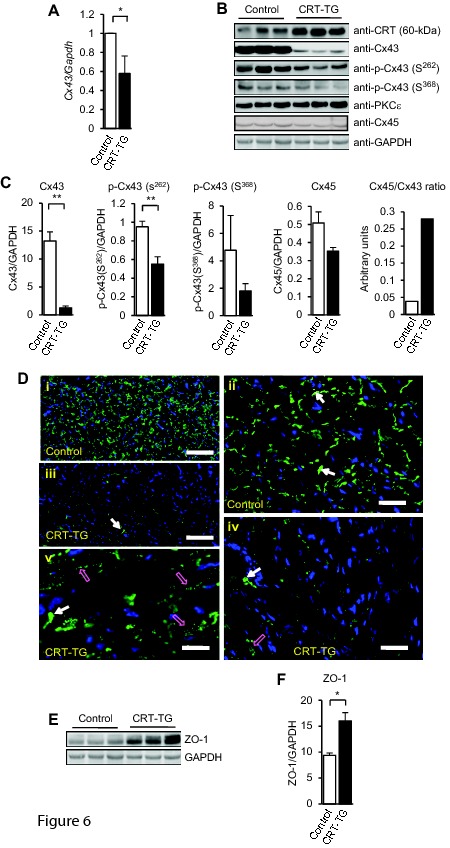


In figure 7C □-gal should be replaced with β-gal in 1 place. Please see the corrected figure 7 here: 

**Figure:**