# Supplementary figures and images for: Calreticulin Induces Dilated Cardiomyopathy
Source: PLoS One. 2013 Feb 20;8(2):e56387. doi: 10.1371/journal.pone.0056387 (PMC3577809; doi:10.1371/journal.pone.0056387)

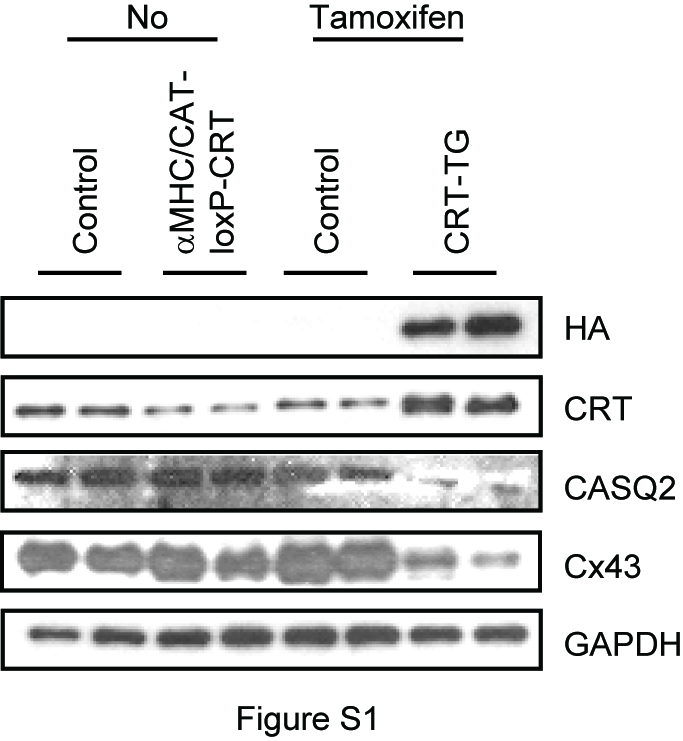

Supplement: Figure S1 — Western blot analysis using HA, CRT, CASQ2, and CX43 antibodies from both control and αMHC/CAT-loxP-CRT heart without tamoxifen and control and αMHC/CRT heart with tamoxifen administration. GAPDH was used as a loading control. HA, hemagglutinin (detect HA-tagged exogenous calreticulin); CRT, calreticulin; CASQ2, cardiac calsequestrin; Cx43, connexin 43. (TIF) [file pone.0056387.s001.tif]
