# Supplementary material for: Calreticulin Induces Dilated Cardiomyopathy
Source: PLoS One. 2013 Feb 20;8(2):e56387. doi: 10.1371/journal.pone.0056387 (PMC3577809; doi:10.1371/journal.pone.0056387)
Supplement: Text S1 — Supporting Information. (DOCX) [file pone.0056387.s003.docx]

**Text S1. Supporting Information**

**Nucleotide sequences *used for real-time* PCR analysis.** CAT, forward: 5’-CAG ACC GTT CAG CTG GA-3’ and reverse: 5’-CAT TCT GCC GAC ATG GAA-3’; MerCreMer, forward: 5’-GTC TGA CTA GGT GTC CTT CT-3’ and reverse: 5’-CGT CCT CCT GCT GGT ATA G-3’; Casq2, forward: 5’-CTT TGC GGA GAA GAG TCA CC-3’ and reverse: 5’-CCA GTC TTC CAG CTC CTC AG-3’; Calm, forward: 5’-TGG CAG TTT GAG TTC GTG AG-3’ and reverse: 5’-CAA GGG CTC AGC AGT TTC TT-3’; CaNA, forward: 5’-ACT GGC ATG CTC CCC AGC GGA-3’ and reverse: 5’-GTG CCG TTA GTC TCT GAG GCG-3’; Mef2c, forward: 5’-AGA TAC CCA CAA CAC ACC ACG CGC C-3’ and reverse: 5’-ATC CTT CAG AGA GTC GCA TGC GCT T-3’; Cx43, forward: 5’-CCT CAC CCT CAC CAA ATG AT-3’ and reverse: 5’- CTG CCT CTG CTT GTA CCA AT-3’; Gapdh, forward: 5’-GTA TGA CTC CAC TCA CGG CAA A-3’ and reverse: 5’-TTC CCA TTC TCG GCC TTG-3’

**The antibodies used for Western blot analysis.** Blots were probed with the following antibodies: mouse anti-HA (hemagglutinin) antibodies (1:500; Santa Cruz Biotechnology), goat anti-calreticulin antibodies (1:500), goat anti-triadin and anti-Junctin antibodies (1:500; Santa Cruz Biotechnology), mouse anti-SERCA2a antibodies (1:1,000; Abcam), rabbit anti-CASQ2 antibodies (1:1,000), rabbit anti-RyR2 antibodies (1:500; Millipore), mouse anti-phospholamban (1:5,000), rabbit anti-phospho (S^16^/T^17^)-phospholamban antibody (1:5000; Cell Signaling), rabbit anti-Cx43 (1:10,000), rabbit anti-phospho (S^262^)-Cx43 antibody (1:2000; Santa Cruz Biotechnology), rabbit anti-phospho (S^368^)-Cx43 antibody (1:2000 dilution; Santa Cruz Biotechnology), anti-Cx45 antibodies (1:1,000; Santa Cruz Biotechnology), mouse anti-NCX1 antibodies (1:200; Abcam), rabbit anti-ZO-1 antibody (1:1000; Life Technologies), and rabbit anti-GAPDH antibody (1:1000 dilution; Abcam).
